# Disseminated inflammation of the central nervous system associated with acute hepatitis E: a case report

**DOI:** 10.1186/s12883-020-01952-5

**Published:** 2020-10-27

**Authors:** Jan Rahmig, Arne Grey, Marco Berning, Jochen Schaefer, Martin Lesser, Heinz Reichmann, Volker Puetz, Kristian Barlinn, Timo Siepmann

**Affiliations:** 1 Department of Neurology, University Hospital Carl Gustav Carus, Technische Universität Dresden, Fetscherstraße 74, 01307 Dresden, Germany; 2 Department of Neuroradiology, University Hospital Carl Gustav Carus, Technische Universität Dresden, Dresden, Germany; 3 Internal Medicine Department I, Gastroenterology, University Hospital Carl Gustav Carus, Technische Universität Dresden, Dresden, Germany

**Keywords:** Neuroinflammation, Ribavarin, Hepatitis E, Encephalitis, Retrobulbar neuritis

## Abstract

**Background:**

Hepatitis E infection affects over 20 million people worldwide. Reports of neurological manifestations are largely limited to the peripheral nervous system. We report a middle-aged genotype 3c male patient with acute hepatitis E virus (HEV) infection and severe neurological deficits with evidence of multiple disseminated inflammatory lesions of the central nervous system.

**Case presentation:**

A 42-year-old male patient presented to our emergency department with musculoskeletal weakness, bladder and bowel retention, blurred vision and ascending hypoesthesia up to the level of T8. Serology showed elevated liver enzymes and positive IgM-titers of hepatitis E. Analysis of cerebrospinal fluid (CSF) showed mild pleocytosis and normal levels of glucose, lactate and protein. HEV-RNA-copies were detected in the CSF and stool. Within 3 days after admission the patient became paraplegic, had complete visual loss and absent pupillary reflexes. MRI showed inflammatory demyelination of the optic nerve sheaths, multiple subcortical brain regions and the spinal cord. Electrophysiology revealed axonal damage of the peroneal nerve on both sides with absent F-waves. Treatment was performed with methylprednisolone, two cycles of plasma exchange (PLEX), one cycle of intravenous immunoglobulins (IVIG) and ribavirin which was used off-label. Liver enzymes normalized after 1 week and serology was negative for HEV-RNA after 3 weeks. Follow-up MRI showed progressive demyelination and new leptomeningeal enhancement at the thoracic spine and cauda equina 4 weeks after admission. Four months later, after rehabilitation was completed, repeated MRI showed gliotic transformation of the spinal cord without signs of an active inflammation. Treatment with rituximab was initiated. The patient remained paraplegic and hypoesthesia had ascended up to T5. Nevertheless, he regained full vision.

**Conclusions:**

Our case indicates a possible association of acute HEV infection with widespread disseminated central nervous system inflammation. Up to now, no specific drugs have been approved for the treatment of acute HEV infection. We treated our patient off-label with ribavirin and escalated immunomodulatory therapy considering clinical progression and the possibility of an autoimmune response targeting nerve cell structures.

While response to treatment was rather limited in our case, detection of HEV in patients with acute neurological deficits might help optimize individual treatment strategies.

## Background

Hepatitis E infection is a highly prevalent viral infection affecting around 20 million people around the globe with an estimated symptomatic course for 3.3 million people [1, 2]. This can be explained by the poor hygiene standards in the Asian and African area and by the pathogenetic high virulence as well as its predominantly faecal-oral path of transmission either from contaminated water or zoonotic transmission [3, 4]. In the western area, hepatitis E virus (HEV) is predominantly transmitted by congestion of raw meat of pork or game [5]. Moreover 15% of native wild boars in Germany are carrier of the hepatitis E virus [6]. Once an infection took place most humans are asymptomatic. Referring to a Chinese vaccination study, less than 5% of patients develop classic signs of an acute hepatitis with fever, stomach pain, nausea, vomiting and became icteric [7, 8]. Neurological complications have been described in up to 10% of symptomatic cases [9]. However, the vast majority of those were limited to neuralgic amyotrophy and myalgia with the exception of a few reports of single cases with Guillain-Barré-Syndrome [10], Neuralgic amyotrophy [9, 11], Bell’s palsy [12], Myasthenia gravis [13] and very rare reports of central nervous system involvement presenting as myelitis or encephalitis [14–16]. Interestingly, all reported patients who experienced neurological deficits during hepatitis E were infected by the specific virus genotype 3 [17]. To the best of our knowledge, we report the first presentation of multiple disseminated inflammatory lesions of the central nervous system with otherwise inscrutable progressive neurological deficits in a patient with acute HEV infection.

## Case presentation

A 42-year-old male patient presented to our emergency department with musculoskeletal weakness, bladder and bowel retention, blurred vision and ascending hypoesthesia up to a level of T8 within 4 days before admission. Clinical examination showed an anicteric, cardiopulmonary stable patient. We saw a patient with spasticity of the lower limbs with a non-exhaustive clonus of the achilles tendon reflex and strength of the lower limbs was found to be 3/5 on the right and 4/5 on the left according to the “British Medical Research Council-Scale” (BMRC) [18]. The upper limbs were clinical unremarkable. Blood samples which were taken on admission showed elevated liver enzymes (alanine-transaminase: 40.16 μmol/(s*l); aspartate-transaminase: 16.45 μmol/(s*l); gamma-glutamyltransferase: 9.71 μmol/(s*l), bilirubin: 31.9 μmol/l). A standard serum viral test battery was performed suspecting an acute viral hepatitis. It includes serology testing of hepatitis A, B, C, D and E. Serology testing revealed an acute HEV infection with a positive titer of IgM and 4.09e+ 02 IU/ml copies of HEV-RNA. Analysis of the CSF showed pleocytosis (15 cells, 43 atypical lymphocytes/mm^3^) with normal levels of glucose, lactate, and protein as well as HEV-RNA copies [333 IU/ml]) without signs of an infection with CMV, VZV, HSV 1, 2, 6 or any other hepatic pathogens. HEV-RNA copies were also found in a stool sample.

Genotyping and RNA quantification were confirmed at German national reference laboratories. Serology was found to be negative for antinuclear and extractable nuclear antigens, anti-ganglioside-, anti-aquaporin-4-, anti-VGCC- and anti-VGKC-antibodies. A test of HIV antibodies was negative. The diagnosis of a parainfectious myelitis caused by a bacterial infection was excluded by negative blood culture test results of streptococcus pneumonia, legionella, treponema pallidum. Within 3 days after admission the patient became paraplegic, had full visual loss, absent pupillary reflexes. The patient described dysesthesia in the arms which ascended within 3 days after admission up to the shoulders.

**Fig. 1:**
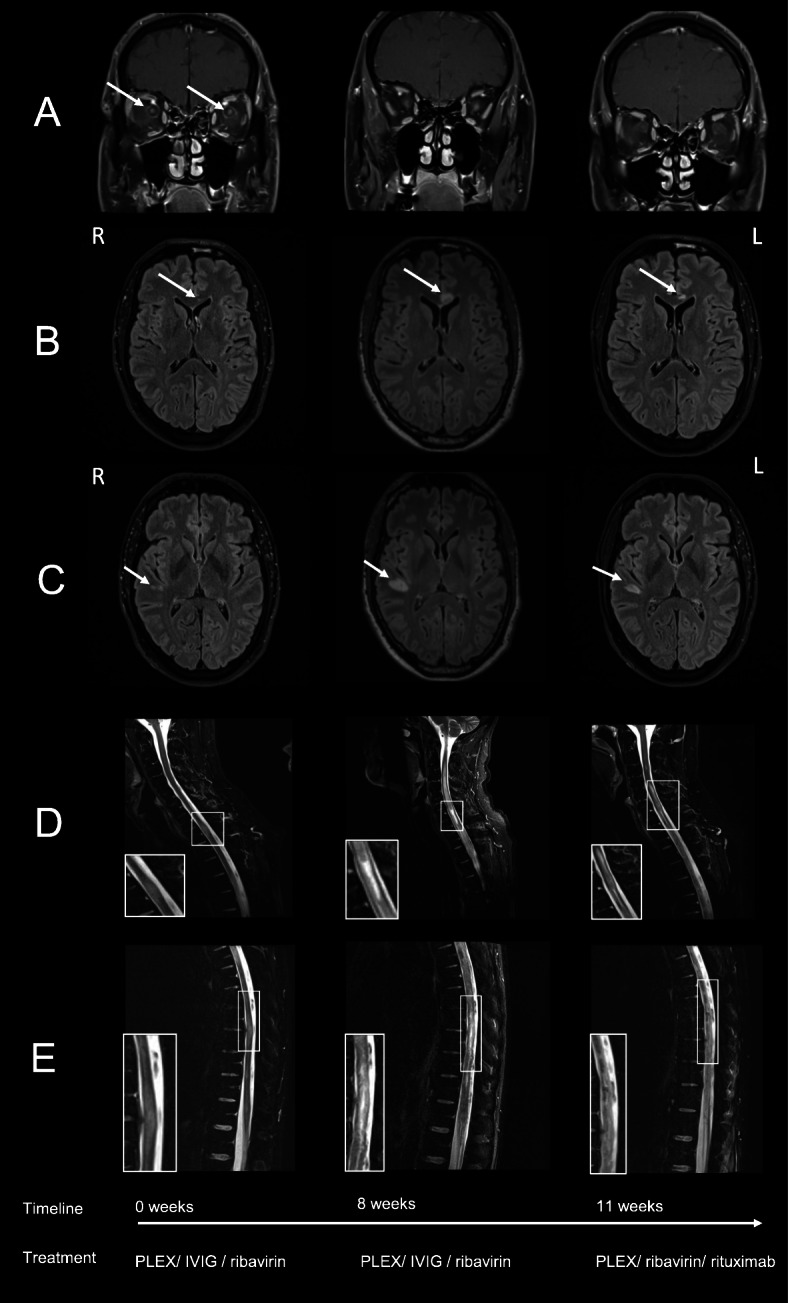
Radiological findings in the course. Brain MRI of hepatits-E-related lesions displayed in a time chart over 11 weeks (from left to right). **a** Retrobulbar enhancement of nervi optici which shows a regredient state, coronal T1-weighted images with contrast medium. **b**, **c** Hypersignal of the brain marrow of the temporal lobe and rostral of the corpus callosum on the left which shows a deterioration in the second picture. After 11 weeks a decline of the lesions were found (FLAIR). **d** A 10 mm hyperintense lesion at the cervical spine was found and declared as a severe edema which caused swelling. The middle picture shows a significant deterioration of the lesion. The right picture shows a partial decline of the lesion (T2, turbo inversion recovery magnitude (TIRM)-weighted images without contrast medium). **e** Significant space-consuming hyperintense lesion of the thoracic spine was detected in the first MRI (left picture). Middle picture shows a distinct progredient edema. The right picture shows a significant regression of the pathologies. (TIRM-weighted images without contrast medium). Investigations were performed with 3.0 Tesla, Siemens Magnetom Verio for the left and right pictures and for the middle pictures: 3.0 T Siemens Magnetom Vida was used

First taken cranial MRI showed demyelination of the white matter adjacent to the lateral ventricle and of the rostral corpus callosum (Fig. 1a). Moreover subcortical T2 and FLAIR hyperintense lesions were found in the superior frontal gyrus on the right and punctual white matter lesion were observed in the precentral right lobe. With the use of contrast medium, bilateral enhancement of the optic nerve sheaths with an extension of 7 mm were detected consistent with a retrobulbar neuritis (Fig. 1b). Spinal T2 weighted MRI showed small monosegmental hyperintensities at the cervical level 7 and thoracic level 2, 5, 8/9 and 12 leading down to the conus medullaris. Lesions appeared unchanged after using a contrast medium (Fig. 1c). Electrophysiology revealed axonal damage of the peroneal nerve on both sides with absent F-waves. Upon confirmation of a HEV infection, treatment with ribavirin (1 g per day) was initiated. Subsequently, liver enzymes normalized after 1 week. Serum HEV-RNA was negative after 3 weeks. In addition, two cycles of plasma exchange (PLEX) and one cycle of intravenous immunoglobulins (IVIG; 0.35 g/kg/d for 10 days) were performed over a period of 11 weeks.

Two weeks after treatment initiation, MRI showed progressive demyelination and new leptomeningeal enhancements at the thoracic spine and cauda equina with hemorrhagic lesions (Fig. 1a-c). Furthermore, we found new turbo inversion recovery magnitude (TIRM)-weighted retrobulbar lesions and after adding contrast medium, small enhancements of the optic nerves were detected. A progression of the lesions in the rostral corpus callosum on the left and the right precentral lobe was detected in T2 and FLAIR-weighted images. A new lesion was observed in the temporal lobe on the right as well as new punctual T2 hyperintensities on the right temporal and the left precentral lobe. An initially observed FLAIR-hyperintense lesion of the superior temporal lobe on the right was stable. The cord spine showed a significant progress of T2 hyperintensities from the cervical level 2 down to the conus medullaris. Within this long stretched lesion, a ten-millimeter long hypointense lesion with adjacent enhancement was detected. As these radiological findings progressed, the patient remained paraplegic and hypoesthesia had ascended up to T5 after completion of treatment. Nevertheless he regained full vision.

Subsequently, the patient had been transferred to a rehabilitation center before he was readmitted to our clinic for follow-up assessment 6 weeks later. Repeated physical examination and electrophysiology revealed no improvement. Follow up MRI showed regressive enhancement of the optic nerve sheaths. A partial decrease of supratentorial white matter T2-hyperintense lesions were observed. There were no new enhancements in the brain or spine. Furthermore, the spinal cord lesions displayed gliotic transformation with reduced caliber and swelling absent any signs of active inflammation.

The patient underwent another cycle of PLEX and rituximab (5 courses; 1 g per day) was added to the regimen. The patient showed less dysesthesia of the arms and shoulders and began to feel tingling in the thighs and the left feet. Power of the limbs remained unchanged and the patient was transferred to continue rehabilitation.

## Discussion and conclusion

Our observation of multiple inflammatory lesions of the central nervous system with severe progressive neurologic deficits in a patient with acute hepatitis E might be relevant to characterizing how HEV affects neural structures. The majority of previous reports indicated that neurologic involvement in patients with acute hepatitis E is largely limited to the peripheral nervous system and occasional associated to seizures without evidence of a structural correlate on imaging studies [9, 10, 14–16]. Our patient showed a severe central nervous system inflammation with multiple disseminated lesions on the cerebral and spinal level, exceeding the extent of neural involvement observed in patients with acute hepatitis A, B, and C infections [19–22].

A possible differential diagnosis acute disseminated encephalomyelitis (ADEM) should be discussed in this case report as it can affect the brain and the spinal cord simultaneously. ADEM is more often affecting children, nevertheless it can also be seen in adults. It is a monophasic acute demyelinating disease, which occurs following an infection, after vaccination or as an idiopathic disease. Patients with ADEM commonly present with varying symptoms according to the structural lesions of the central or peripheral nervous system. In particular, widespread, multifocal, or extensive white matter lesions accounting for up to 50% of the entire white matter are frequently observed. Moreover, lesions in the deep grey matter are commonly seen in ADEM. Areas of the thalamus or the basal ganglia can be affected, often bilaterally [23]. In contrast, our patient just showed mild disseminated bilateral lesions without affecting the grey matter. Furthermore, CSF-associated myelin autoantigens such as myelin basic protein-, proteolipid protein-, and myelin oligodendrocyte (MOG) protein-antibodies can be found in patients with ADEM [24]. Anti-viral antibodies or a cell-mediated response to the pathogen cross-react with the myelin autoantigens, resulting in ADEM.

However, our patient did not show anti-MOG-antibodies or oligoclonal bands and neurological deficits as well as structural lesions occurred and progressed during the course of an acute hepatitis E infection. The vast majority of patients with ADEM recover over a period of 1–6 months with the help of corticosteroids or IVIG even though it has been described that residual deficits or even death is a possible outcome in previous case reports [23, 25, 26]. Steroids and IVIG were used in our patient with little effect. Therefore, we escalated therapy to plasmapheresis and rituximab, which also showed little effect. In conclusion, we saw a patient with disseminated white matter brain lesions that were not consistent with a diagnosis of ADEM. However a rare manifestation of ADEM could not be excluded entirely.

The leading diagnosis to the best of our knowledge is an acute disseminated encephalomyelitis with bilaterally retrobulbar neuritis and extensively cord affection, possibly associated with an acute HEV-infection.

In fact, due to the progressive course and the disseminated pattern of neuronal damage in our patient, we initially considered acute haemorrhagic leucencephalitis (Weston-Hurst-Syndrome, AHLE), a fulminant and rare entity of an acute disseminated encephalomyelitis (ADEM). However, in contrast to our patient, MRI in AHLE patients commonly reveal large patches of demyelination in the white matter, haemorrhages and edema with space occupying effect. Moreover, frequently seen characteristic of AHLE seems to be a monophasic clinical course with a fulminant high mortality rate. Most patients were dying within 2–4 days of disease onset [27, 28]. Even though, the pathogenesis still remains unknown an underlying autoimmune aetiology is suspected. In our patient, relatively slow clinical and radiological progress of lesions was particularly not consistent with AHLE [29].

Initially, we in fact considered the possibility of Devic’s-syndrome. However, in patients with Devic’s-syndrome brain MRI is commonly normal. Brain lesions may occur in patients with neuromyelitis optica spectrum disorder (NMOSD) typically, but not always, in later phases of the disease. By contrast, our patient showed multiple lesions in the MRI. Furthermore, spinal fluid analysis does not usually show oligoclonal bands in patients with Devic’s-syndrome or NMOSD and MOG-IgG, is present in about half of those who do not have AQP4-IgG. Our patient showed neither the one nor the other in both serology and liquor analyses. With negative test results of aquaporine-4-antibodies plus the incoherent patterns of lesions, especially extensive lesions down to the conus medullaris, we excluded Devic’s-syndrome or NMOSD as a leading differential diagnosis. Another rational for ruling out Devic’s-syndrome was a missing response to pulsed methylprednisolone therapy. Consistently, plasmapheresis had little effect on the clinical course.

Our literature search revealed a previous report of a possible association of acute HEV infection with spinal demyelination. This case report described a 62-year-old Caucasian woman who suffered paraplegia associated with an acute HEV infection [16]. Within 1 day after admission power of her lower limbs was found to be 0/5 according to BMRC. She showed sensory loss up to a level of T8. Treatment contained of two courses of pulsed methylprednisolone. MRI showed no signs of a spinal cord compression or cauda equina syndrome. Response to treatment had not been reported and observational studies on this possible pathophysiological link are still lacking. In contrast to this report, in our patient, infection did not exclusively affect the cord spine but also comprised a retrobulbar neuritis and an encephalomyelitis with widespread cerebral lesions leading to severe neurological impairment.

Another case was published in 2006 describing a 12-year-old Indian girl who developed a transverse myelitis followed by an acute HEV infection [15, 16]. The young girl presented with a cord syndrome and a sudden onset of weakness of both lower limbs. MRI showed a swelling of the cervical spine cord with a hyperintensity. CSF revealed a normal test result. Serology for HEV IgM was positive. She recovered spontaneously within 2 days after admission using conservative treatment [15]. By contrast, we treated our patient with methylprednisolone, PLEX and IVIG due to the suspicion of an immune mediated pathological pathway affecting nerve cell structures. Normally, no treatment of immunocompetent patients with an acute hepatitis E virus infection is needed due to a high self-curing-rate.

There are different hypotheses of suspected mechanisms how HEV affect nerval structures and infect them. Invasion may result in unspecific binding of the virus via membrane cloaking as a direct mechanism [30]. In one described case of detected HEV in the CSF a different viral sequence compared to circulating HEV-RNA in the blood was found. The reason for that still remains unknown and needs deeper sequencing to assess the diversity of the virus [31]. Possible indirect mechanisms include cross-reactive immune responses postinfectious or the reactivation of a latent virus or a suspected coinfection by other pathogenic microbes due to an immunocompromised patient [32]. However due to a substantial lack of data these possible pathophysiological pathways remain speculative. It needs to be emphasized that an evidence-based therapy for hepatitis E infection is not available to date. While ribavirin was suggested to be an effective treatment in patients with hepatitis A, B and C, only single case reports support a decrease of viremia in acute hepatitis E virus infection [33–37]. We based our decision using off-label treatment on the severity of neurologic deficits and structural damage to the CNS. For the same reason, we chose to escalate the treatment regimen using PLEX, IVIG and rituximab due to clinical progression and the possibility of an autoimmune-mediated association between HEV and nerve cell structures [17].

In conclusion, detection of HEV in patients with acute neurological deficits and elevated liver enzymes might be helpful in optimizing individual treatment strategies. Our patient differs from previous reports of HEV related neuroinflammation. In fact, central involvement with disseminated demyelination of the brain marrow, the cord spine and a bilateral retrobulbar neuritis viewed in conjunction with elevated liver enzymes and positive IgM-Titers of HEV might indicate that HEV-related neuroinflammation can exceed pure PNS involvement. In patients with elevated liver enzymes and positive IgM-Titers of HEV presenting with neurological deficits of unidentifiable cause, HEV-associated neuroinflammation might be a valid differential diagnosis. Therefore, observational and meta-analytic research is urgently needed to elucidate the underlying pathophysiology and identify therapeutic targets.

## Data Availability

The datasets used and/or analyzed during the current study are available from the corresponding author on reasonable request.

## Abbreviations

AHLE: Acute haemorrhagic leucencephalitis
ADEM: Acute disseminated encephalomyelitis
BMRC: British Medical Research Council-Scale
CMV: Cytomegalovirus
CNS: Central nervous system
CSF: Cerebrospinal fluid
HEV: Hepatitis E virus
HSV: Herpes simplex virus
IVIG: Intravenous immunoglobulin
MRI: Magnetic resonance imaging
MOG: IgG- Myelin oligodendrocyte glycoprotein
NMO: Neuromyelitis optica
NMOSD: Neuromyelitis optica spectrum disease
PLEX: Plasma exchange
TIRM: T2, turbo inversion recovery magnitude
VZV: Varicella zoster virus
VGCC: Voltage-gated calcium channel
VGKC: Voltage-gated potassium channel antibodies
